# Contributions of HLA haplotypes, IL8 level and *Toxoplasma gondii* infection in defining celiac disease's phenotypes

**DOI:** 10.1186/s12876-018-0796-9

**Published:** 2018-05-18

**Authors:** Mohammad Rostami-Nejad, Seyed Hossein Hejazi, Amado Salvador Peña, Hamid Asadzadeh-Aghdaei, Kamran Rostami, Umberto Volta, Mohammad Reza Zali

**Affiliations:** 1 Celiac Disease Department, Gastroenterology and Liver Diseases Research Center, Research Institute for Gastroenterology and Liver Diseases, Shahid Beheshti University of Medical Sciences, Tehran, Iran; 2 Skin Diseases and Leishmaniasis Research Center, Isfahan University of Medical sciences, Isfahan, Iran; 3 Laboratory of Immunogenetics, Department of Medical Microbiology and Infection Control, Vrije Universiteit Medical Center (VUmc), Amsterdam, the Netherlands; 4 Basic and Molecular Epidemiology of Gastrointestinal Disorders Research Center, Research institute for Gastroenterology and Liver Diseases, Shahid Beheshti University of Medical Sciences, Tehran, Iran; 5 Department of Gastroenterology, Milton Keynes University Hospital, Milton Keynes, UK; 6 Department of Medical and Surgical Sciences, University of Bologna, Bologna, Italy

**Keywords:** Celiac disease, Toxoplasmosis, Antibodies, HLA alleles, Histological features, Gluten

## Abstract

**Background:**

It is not clear why some patients with coeliac disease (CD) present with severe symptoms and small intestinal mucosal damages while others present with milder symptoms and no frank enteropathy. There is no study to assess the associated factors with mild/severe symptoms and enteropathy. The terminologies like latent, silent and potential are difficult to use and are unrepresentative. In the present study we describe coeliac disease’s phenotypes based on HLA haplotypes, IL8 production and past infection with *Toxoplasma gondii* (*T. gondii*) infection*.*

**Methods:**

In this case-control study, sera originating from 150 healthy subjects and 150 patients diagnosed with CD during the years 2013–14 were analyzed for the presence of antibodies specific *T. gondii* of the IgG and IgM subclasses. The level of IL8 were measured and HLA-DQ2 and HLA-DQ8 alleles were genotyped. The correlation between these parameters and the damages in intestinal mucosal were assessed using an accepted histopathological classification.

**Results:**

High levels of IgG antibodies against *T. gondii* were found in the sera of control group compared to the CD group (52.6% vs. 39.4%, *P* = 0.02). Mean serum levels of IL8 was significantly higher in CD patients compared with control (*P* ≤ 0.05). By comparing the level of anti- *T. gondii* IgG and mucosal damage in celiac disease, we found a significant relationship between the severity of mucosal damages and anti- *T. gondii* IgG level (*P* = 0.02). No correlation was detected between *Toxoplasma gondii* infection and types of HLA (*P* > 0.05). However, patients with severely abnormal histology carried HLA-DQ2 risk alleles (92 patients (61%)) more often than the controls and those with mild histological abnormalities.

**Conclusions:**

CD patients with severe histological changes had more often *Toxoplasma gondii* infection than those affected with mild histological features. This suggests that CD’s phenotypes are correlated to additional factors like infections and to particular HLA DQ2 alleles that may need additional investigations and potentially will require additional treatment.

**Electronic supplementary material:**

The online version of this article (10.1186/s12876-018-0796-9) contains supplementary material, which is available to authorized users.

## Background

The spectrum of histological abnormalities in CD ranges from mild to severe mucosal changes however, the correlation with the symptoms of the patients is poor [1, 2]. The classical presentation is becoming extremely rare and the majority of the patients have an atypical presentation [1]. The terminology used in the classification of CD, like silent, potential and latent, fails to distinguish effectively between atypical forms of this condition and often leads to the confusion of the clinicians [2]. Subsequently many patients are left in uncertainty without treatment. The differences in small bowel permeability, caused by different degrees of mucosal abnormalities leading to a variety of intestinal or extraintestinal symptoms, result in different disease phenotypes. The innate and adoptive immunities by inflammatory and pro-inflammatory cytokines like IL1, IL6, IL8, and IL17 induce the inflammation in the small bowel mucosa that leading to mild to severe malabsorption syndrome [3].

More than 90% of celiac disease patients were HLA-DQ2 positive [4]. CD may affect patients at any age, and the intestinal lesions may be accompanied by abnormalities in other organs with manifestations or complications, including brain, mood and reproductive organs [5]. The factors associated with these associations have not been studied adequately. How far gluten is involved in the pathogenesis of neurological disorders such as autism, schizophrenia, mental retardation and bipolar disorder is not elucidated [5–7]. In contrast to other autoimmune conditions, where the trigger of the disease is not known, [8] the strict removal of the gluten of the diet reverts the small intestinal abnormalities to normal and it is effective in treatment of some associated conditions such as dermatitis herpetiformis and some of the patients with gluten ataxia. The lack of correlation between the symptoms and histological changes induces the searching of other factors to understand the clinical significance and the pathogenesis of the disease.

According to the result of animal studies, as soon as *Toxoplasma gondii* invades the epithelial cells of the small intestine a severe form of intestinal inflammation is induced [9–11].

In the small intestine, *Toxoplasma gondii* infection may initiate the sequence of innate immunologic reactions that usually result in a vigorous inflammatory process [11]. In the mice model, the immune reaction result in morphologic and histologic characteristics similar to human intestinal autoimmune disorders such as celiac disease and inflammatory bowel disease like destruction of intestinal epithelial cells, shortened villi, substantial invasion of inflammatory cells into the lamina propria and an increase number of intraepithelial lymphocytes (IELs) [12].

In addition to environmental factors such as infectious agents and diet and immunological issues like cytokines, genetic influence mostly by the human leukocyte antigens HLA DQ2 and DQ8 are necessary for CD development. We hypotheses that the combination of *T. gondii* infection, HLA predisposition and inflammatory cytokines like IL8 may contribute in histological abnormality and their presence may introduce as representative marker of mucosal severity of celiac disease patients.

Therefore, the aim of this study was to evaluate the role of the presence of *Toxoplasma gondii* previous infection, HLA alleles, and IL8 level at the time of presentation in the severity of the mucosal damage and their possible impact on CD phenotype.

## Methods

### Patients

One hundred and fifty untreated celiac disease patients with serum anti-human tissue Transglutaminase (tTG) IgA positive test and histopathological evidence according to the Marsh classification were randomly enrolled following referral to the celiac disease department unit of Research Institute for Gastroenterology and Liver Diseases, Shahid Beheshti University of Medical Sciences, Tehran, Iran during the 2012–2014. The 150 healthy subjects without CD matched by age were selected from referred cases to Taleghani hospital’s lab for routine blood test.

A questionnaire covering demographic data, patients’ symptoms and medication used was filled in. Four biopsies were taken from the second portion of the duodenum, one from bulb and one from D3 [13] oriented on filter paper, fixed overnight in buffered formalin, embedded in paraffin, cut to 3-μm thickness, and stained with hematoxylin-eosin for routine histological evaluation.

The Hematoxylin and Eosin (H&E) slides were reviewed by expert pathologists and histopathological findings were evaluated in according to the most recently revised Marsh classification [12] as presented and accepted during the International celiac disease congress, Prague 2015 [14].

Informed consent was obtained from each patient prior to study enrollment. The study was approved by ethics review board of Gastroenterology and Liver Diseases Research Center, Shahid Beheshti University of Medical Sciences, Tehran, Iran (IR.SBMU.RIGLD.1395.90).

### Blood sample collection

Ten milliliters of venous blood was drawn for both specific celiac disease antibodies and *Toxoplasma gondii* antibodies and sera were separated and kept at − 70 °C. tTG-IgA antibody was measured using a commercially available ELISA kit (AESKULISA tTGA, Germany) according to the manufacturer’s guidelines and the result was considered positive when a value higher than 15.0 U/ml was recorded. Total serum IgA values were measured by an immunoturbidometric assay (Pars Azmoon, Iran) and according to manufacturer’s guideline, serum levels below 70 U/L were considered indicative of IgA deficiency.

Immunoglobulin G (IgG) tTG values were further obtained in individuals with IgA deficiency by an ELISA method, and using the commercially available kit AESKULISA tTGG (Germany).

In addition, the *Toxoplasma* specific IgG and specific IgM antibodies in the same serum samples were assayed by commercial ELISA kit (ELISA, Vircell *Toxoplasma* IgG&IgM/Spain) according to the manufacturer’s instruction. In keeping with the manufacturer’s guidelines, a result was considered positive when a value higher than 1:10 IU/ml was recorded.

IL8 level was measured according to the manufacturer’s instruction in both groups by commercial Enzyme Linked Immunosorbant Assay kit (Human IL8/NAP-1 ELISA, Bender MedSystems, Austria).

We considered a diagnosis of CD if the patients had high levels of IgA anti-tTG > 3 times of upper normal limit and confirmed by appropriate histology and HLA typing. For instance in patients with a phenotype of Marsh I-II, the diagnosis was confirmed as potential celiac disease (PCD) by HLA typing and high titers of IgA anti-tTG, 3 times higher than cut off or positive at any titer, but confirmed by IgA EMA positivity.

### HLA typing

For HLA typing genomic DNA was extracted using salting out method and HLA-DQ2/HLA-DQ8 haplotypes were genotyped by Real-time PCR using SYBR Green as described [15]. In brief, HLA-DQB1*02 was determined for HLA-DQ2, HLADQB1* 02 and HLA-DQA1*05 for HLA-DQ2.5 and HLA-DQB1*0302 for HLA-DQ 8.

### Statistical analysis

Percentages were compared by rates and proportion; 95% confidence intervals are reported. We also used the corrected χ^2^ test or Fisher’s exact test and odds ratio to compare percentages, and the unpaired Student t-test to compare the means of normally distributed variables. *Tukey’s Multiple Comparison Test* and *Multivariate logistic regression* analysis was performed to analysis the difference between groups.

## Results

One hundred and fifty subjects with CD (mean age ± SD = 34.9 ± 13.68) were recruited as case and 150 individuals (mean age ± SD = 34.1 ± 13.95) as control. No statistically significant differences were noted between the groups regarding demographic characteristics. The main symptoms of celiac disease at the time of diagnosis was diarrhea [45/150 cases (30%)], anemia [26/150 subjects (17.3%) and epigastric pain [22/150 individuals (14.7%)] respectively. Between the extra intestinal presentations menarche disturbances (4.4%) and abortion (6%) were low prevalent. In patients with Marsh I, 22 (73%), presented with weight loss, 20 (67%) with neurological disorders and bloating/osteopenia in 16 (53.3%) and in patients with Marsh II, bloating 23 (65.7%), osteopenia/neurological disorders 21 (60%), and weight loss 20 (57.1%) were found most prevalent. Similarly in patients with Marsh III, weight loss [63 (74.1%)], bloating [58 (68.2%)] and osteopenia in 55 (64.7%) were recorded. Statistical analysis revealed no significant relationship between histology and gastrointestinal symptoms among celiac disease patients (Table 1).

**Table 1:** Relationship between Marsh classification and gastrointestinal symptoms and signs in patients with celiac disease

Patient’s symptoms and signs	Marsh classification (%)			*P* value
	Marsh I	Marsh II	Marsh III	
Diarrhea	9 (30)	8 (22)	30 (35.9)	0.39
Nausea and vomiting	10 (34)	14(40)	29 (34.1)	0.8
Weight loss	22 (73)	20 (57.1)	63 (74.1)	0.16
Heart burn	15 (50)	21 (60)	52 (61.1)	0.55
Bloating	16 (53.3)	23 (65.7)	58 (68.2)	0.34
Anemia	5 (17)	12 (34.3)	24 (28.2)	0.24
Osteopenia	16 (53.3)	21 (60)	55 (64.7)	0.65
Neurological signs	20 (67)	21 (60)	51 (60)	0.79
Menarche problem	0	1 (2.9)	0	0.58
Infertility	0	0	2 (2.3)	0.64
Abortion	1 (3.4)	2 (5.7)	6 (7)	0.7
Aphthous stomatitis	2 (6.6)	3 (8.6)	16 (8.8)	0.22

Fifty nine (39.4%) of patients with CD and 79 (52.6%) of controls, had positive total IgG for *Toxoplasma gondii*. On the other hand, only 0.6% of cases (1 case) and 1.4% of controls (2 subjects) were serologically positive for anti-*Toxoplasma* IgM. There were statistical significant differences between the cases and control regarding total IgG level (*P* = 0.02). There was no significant relationship between age, marital status, employment status, ethnicity, educational status, smoking, history of breast-feeding in infancy and anti-*T. Toxoplasma gondii* IgG in celiac patients compare to controls (*P* > 0.05). Also no significant associations were observed between type of meat consumption, cat keeping and traveling and infection with anti- *Toxoplasma gondii* IgG in two groups (*P* > 0.05).

No statistically significant differences were noted for distribution of either CD or *Toxoplasma gondii* positive patients between different ethnic groups. Compared to anti- *Toxoplasma gondii* IgG negative patients, the level of anti-*Toxoplasma gondii* IgG in patients with bloating was slightly higher (Table 2).

**Table 2:** Relationship between anti-*Toxoplasma* IgG and gastrointestinal symptoms and signs in celiac disease

Patient’s symptoms and signs	IgG		
	Positive (%)	Negative (%)	*P* value
Diarrhea	19 (32.2)	28 (30.8)	0.859
Nausea and vomiting	18 (30.5)	35 (38.5)	0.383
Weight loss	41 (69.5)	64 (70.3)	1
Heart burn	30 (50.8)	58 (63.7)	0.129
Bloating	40 (67.8)	18 (30.5)	0.6
Anemia	18 (30.5)	23 (25.3)	0.346
Osteopenia	33 (55.9)	59 (64.8)	0.543
Neurological signs	36 (61)	56 (61.5)	1
Menarche disturbances	0	4 (4.4)	0.154
Infertility	6 (4)	10 (6.7)	0.332
Abortion	3 (5.11)	6 (6.6)	0.476
Aphthous stomatitis	11 (18.6)	10 (11)	0.233

Both *Toxoplasma gondii* infection and celiac disease are reported to be associated with abortion. In our cohort, 3 out of 9 celiac subjects who had a history of abortion had positive IgG for *Toxoplasma gondii*. Also there was no correlation between symptoms and *Toxoplasma gondii* infection in coeliac patient (*P* > 0.05).

**Fig. 1:**
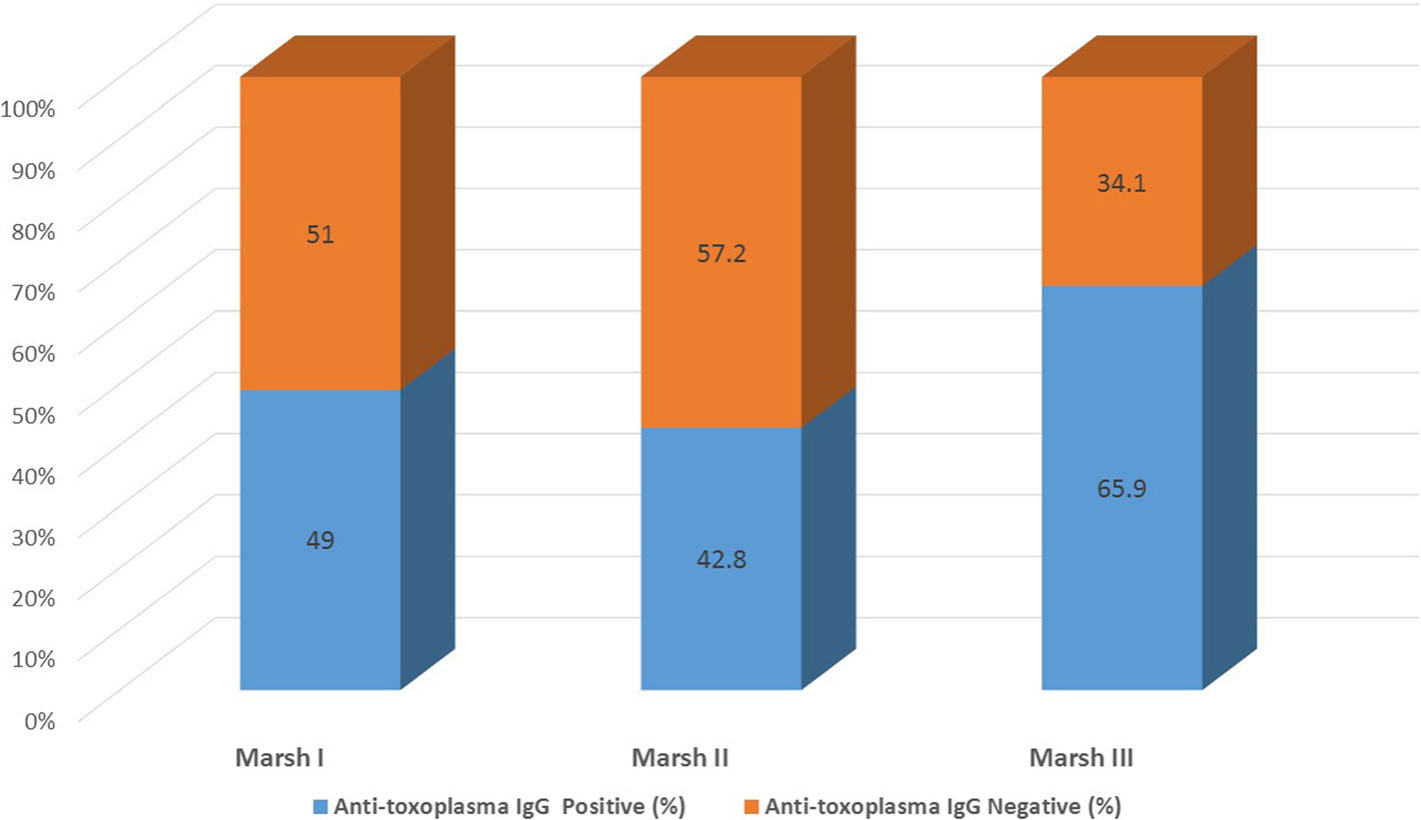
The Relationship between positive and negative levels of IgG anti-Toxoplasma and mucosal damage according to Marsh classification. This differences for Marsh III is statistically significant (*P* Value = 0.02)

By comparing the level of anti-*Toxoplasma gondii* IgG and mucosal damage in celiac disease, we found a significant relationship between the severity of mucosal damages and a positive anti-*Toxoplasma gondii* IgG (*P* = 0.02). Patients with severe mucosal damage such as Marsh III had higher antibody levels than patients with less severe mucosal damage such as Marsh I and II. There was a significant correlation (*P* = 0.05) between positive and negative levels of IgG and mucosal damages as illustrated in Fig. 1.

Serum IL8 levels were evaluated in patients with CD compared with healthy control and the results showed that mean serum levels of IL8 was significantly higher in CD patients (mean serum level ± SD = 176.4 ± 191.8) compared with control (mean serum level ± SD = 68.87 ± 86.8) (*p* = 0.001). There is statistically significant differences between the average level of IL8 in 59 patients with CD and 79 healthy subjects who were positive for anti- *Toxoplasma gondii* IgG [170.2 (SD = 186.66) vs. 66.53 (SD = 87/88)] respectively (*P* = 0.002). But no significant relationship were observed between the level of mucosal damage and IL8 levels in CD patients (*p* = 0.97). Also the level of IL8 in coeliacs with and without *Toxoplasma gondii* infection was not statistically significant (*P* = 0.09).

On the other hand the results showed that in CD patients 77.3% were DQ2 positive, 20% DQ8 positive and 2.7% were negative for both haplotypes. Of these 4 HLA negative patients 2 were Marsh II and 2 Marsh III but all were positive for anti-tTG IgA and anti-EMA IgA with titer 3 times higher than the cut off. For the control group 31.3 and 10% were DQ2 and DQ8 positive respectively and 58.7% were DQ2/8 negative. In the cases 54 patients with Marsh I and II (23 patients with Marsh I and 31 with Marsh II) compared to 92 patients with Marsh III were carried the HLA-DQ2/8 haplotypes but no statistically significant difference was detected between HLA typing and severity of histology (*P* = 0.68). In addition there was no correlation between anti-*Toxoplasma* IgG and HLA typing in either cases or controls (*P* > 0.05). Multivariate logistic regression analysis showed that there is no association between the combination of *T. gondii* infection, HLA predisposition, and IL8 level and mucosal abnormality in CD patients (*p* = 0.17).

## Discussion

Atypical presentation makes the diagnosis of celiac disease very challenging. Although terminologies like latent, silent and potential have been useful in research leading to identify the behavior of some atypical cases with CD, but in clinical practice not only they didn’t simplify the recognition of atypical forms of disease, also contributed to more confusion between both clinicians and patients. By using these terminologies, it is difficult to reach a meaningful diagnosis and clear guide to treatment. The heterogeneity of presentation of CD requires a better classification that would satisfy patients and clinician’s need for an effective diagnosis and a clear guide to management. The cases in this study have been identified using their generic phenotypes.

The question that hasn’t been answered in the current literature is why some patients present with milder histology and others with more severe form of mucosal abnormalities and yet there is no correlation between histology, malabsorption syndrome and symptoms. In this study we have identified some factors that may correlate and explain the variability in presentation of CD. Immune intolerance in celiac disease is connected to genetic (types of genes on chromosomes 5, 6, and 19), and environmental factors such as viral, bacterial or parasitic infections and food antigens. These factors in combination may cause more severe tissue inflammation following losing the tolerance to gluten [16]. See Additional file 1. Review of published relevant studies indicate that infectious agents can contribute to the development of CD in susceptible individuals [17].

In several studies, an association has been found between celiac disease and infections like hepatitis C virus, *adenovirus,* [16, 17] *rotavirus* and *reovirus* [18]. The parasitic infectious agents such as *Giardia lamblia* had been described in several case reports [19]. It looks like the activation of other part of immune system may lead to a more severe mucosal damages and possible additional symptoms in only some cases. For instance in response to parasitic infection, Dendritic Cells (DCs), PMNs specially neutrophils, and macrophage produce different cytokines and chemokines such as IL12, IL8, IL1, IL6, TNFα and INFγ which contribute to the development of the inflammatory response by the direct activation of multiple apoptotic pathways [20, 21]. DCs as main antigen presenting cell has central role in the development of the T cell responses in the lamina propria in the host susceptible to celiac disease and/or parasite infection [22]. We still do not have explanation for why some patients with a phenotype of severe mucosal damages have no much clinical symptoms? [23]. This might be related to other unknown non HLA genetic and/or environmental factors that need further assessments. The result of this study showed that most of the CD patients even positive for *T. gondii* carried HLA DQ2 haplotypes (> 60%) while most of the control were negative for both HLADQ2 and DQ8 haplotypes (> 55%), but there was no correlation between anti-*Toxoplasma* IgG and HLA typing in either cases or controls.

*Toxoplasma gondii* is an intracellular parasite and following ingestion, oocysts are released into the GI tract and tachyzoites enter the host via the small bowel epithelium and then transform into bradyzoites [24].

**Fig. 2:**
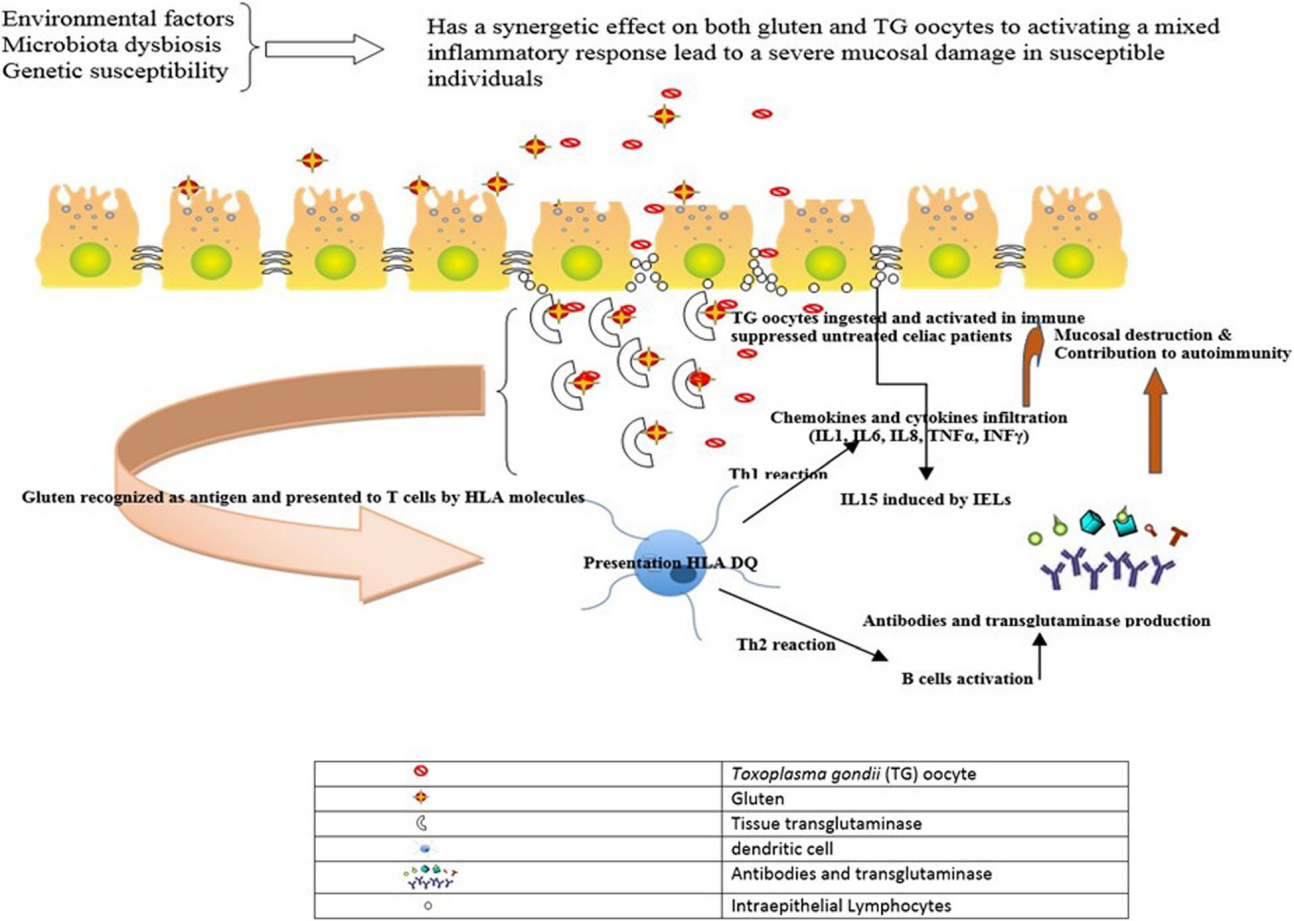
*T. gondii* oocytes are released, penetrating the intestinal epithelium in patients with mild intestinal inflammation caused by gluten. Both are presented to the APC (HLA) and stimulate a Th1 response such as abundant amounts of cytokines such as IL1, IL6, IL8, TNFα and INFγ, neutrophlis, macrophages as well as a Th2 response with production of antibodies against tTG2 and finally activate the adaptative immune response and facilitate the destruction of the villi. Ingested *T. gondii* oocysts probably increase the tissue damage of suffering from celiac disease

Following oral ingestion *T. gondii* in animal model, the small intestine endothelial and epithelial cells produce different types of chemokines and cytokines and CD4+ T cell induce IFN-γ and TNF-α production that will result in flat villi [25]. An experimental study showed a noticeable rise in the intestinal intraepithelial lymphocytes (IELs) population following infection with *T. gondii* similar to celiac disease (10). On the other hands, it may suggest that local microbiota play a critical role in the expansion of inflammatory response activities [26, 27] (Fig. 2).

A few studies have investigated the correlation between celiac disease and *T. gondii* infection [24, 28]. In a study by Plot and colleagues, prevalence of anti-*Toxoplasma gondii* antibodies was higher in control group compared to celiac group (25.9% vs 23.3%). Our results although is compatible with their findings but they did not investigate a correlation between *T. gondii* infection and mucosal abnormality as we done. In our previous study in 2011, of 827 pregnant women, 16 out of 27 (59%) tTGA positive patients were seropositive for *Toxoplasma gondii* infection [24], but we did not evaluate the control group.

## Conclusions

Finally, while the prevalence of anti- *T. gondii* IgG positivity is lower in CD than controls, our results indicate that *T. gondii* infection, increases the risk of severity of histology in celiac disease patients. It would be interesting to compare the intestinal mucosal abnormalities of non-coeliac patients presenting with high anti- *T.gondii* IgG with coeliac disease. Intestinal infections have been involved in triggering celiac disease and clearly *Toxoplasma gondii* may trigger the development of celiac disease in susceptible individuals.

## Additional file

Additional file 1: Factors in combination may cause more severe tissue mucosal damage. TG = *Toxoplasma gondii*. (JPG 48 kb)

## Abbreviations

CD: Coeliac disease
DCs: Dendritic Cells
H&E: Hematoxylin and Eosin
IELs: Intraepithelial lymphocytes
IgG: Immunoglobulin G
NCGS: non-coeliac gluten sensitivity
PCD: Potential celiac disease
*T. gondii*: *Toxoplasma gondii*
tTG: Tissue Transglutaminase
