# Supplementary figures and images for: Contributions of HLA haplotypes, IL8 level and Toxoplasma gondii infection in defining celiac disease's phenotypes
Source: BMC Gastroenterol. 2018 May 18;18:66. doi: 10.1186/s12876-018-0796-9 (PMC5960085; doi:10.1186/s12876-018-0796-9)

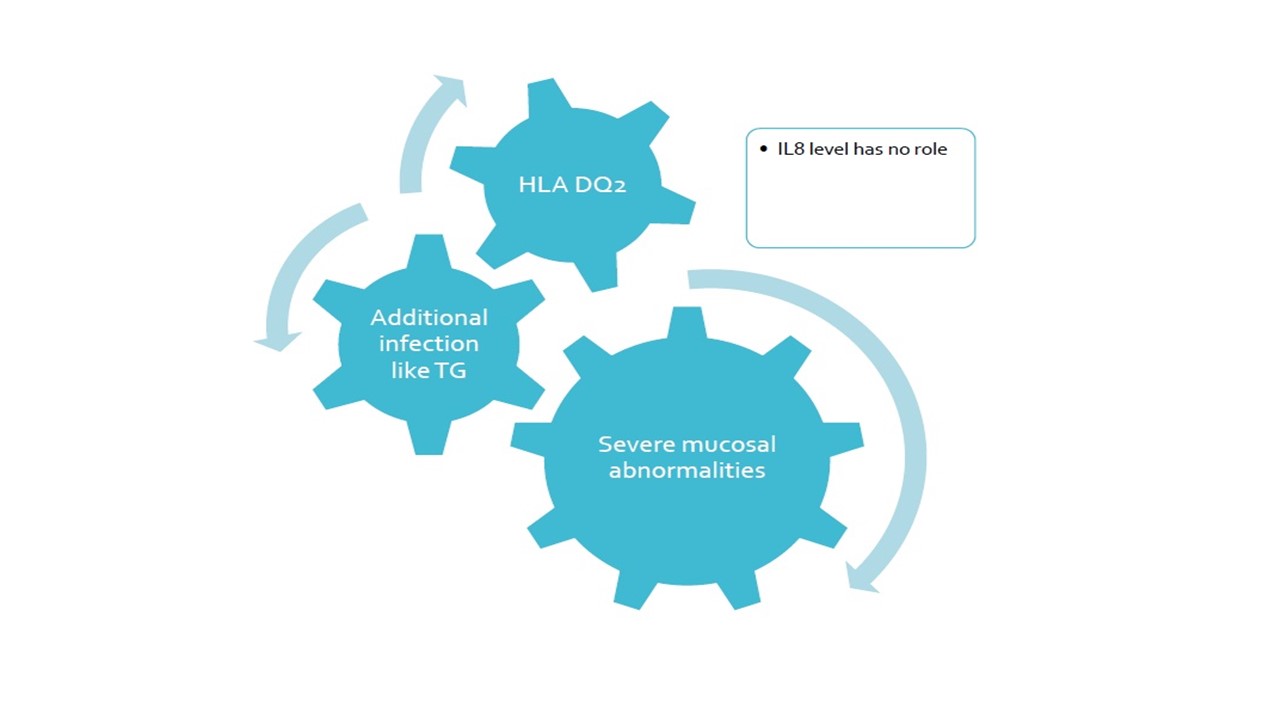

Supplement: Supplementary file 1 — Factors in combination may cause more severe tissue mucosal damage. TG = Toxoplasma gondii. (JPG 48 kb) [file 12876_2018_796_MOESM1_ESM.jpg]
